# Novel Use of Ophthalmic pH Paper to Diagnose Malicious Caustic Ingestion in a Pediatric Patient

**DOI:** 10.5811/cpcem.2017.11.36367

**Published:** 2018-01-09

**Authors:** Neal P. Johnson, Eric C. Bruno

**Affiliations:** University of Tennessee College of Medicine, Saint Thomas Rutherford Hospital, Department of Emergency Medicine, Murfreesboro, Tennessee

## Abstract

Occult caustic ingestion in the pediatric population is a challenging diagnosis to make in the emergency department. Failure to suspect and diagnose a caustic ingestion can lead to potentially life-changing comorbidities. Historically, the diagnosis of caustic ingestion has been clinical without any suitable diagnostic tools to aid in the suspicion of occult cases. In this case, we describe a novel use of ophthalmic pH paper to diagnose caustic ingestion in a three-year-old.

## INTRODUCTION

According to the National Poison Data System, in 2015 there were 2.2 million potentially toxic exposures reported to United States poison centers; of those, about half were among children less than five years of age.^1^ The clinical gestalt of the emergency physician may be the only hope a child has in the setting of a malicious ingestion. Recognition and intervention are critical to preventing acute and chronic life-changing morbidities. We present an innovative way of using ophthalmic pH paper to identify caustic ingestion in a three-year-old boy.

## CASE REPORT

A three-year-old male with a significant past medical history of speech delay presented to an urban emergency department (ED) via ambulance with symptoms of facial edema and erythema. Before the patient’s arrival, the emergency medical services (EMS) providers relayed through the medical communications center that the patient was en route and symptoms were consistent with an acute allergic reaction, likely due to first exposure to Chinese food and dark chocolate that day.

Upon arrival, the patient had right facial erythema with lingual edema and erythema. There was mild pharyngeal erythema without pharyngeal edema or stridor. Initial vital signs were heart rate 142 beats per minute, temperature 36.2 degrees Celsius, respirations 26 per minute, and oxygen saturation 100% on room air. A blood pressure was not recorded. Prior to the onset of symptoms, the patient was not taking any routine medications, nor had he been exposed to new medications. Based on exam and history provided by the mother, maternal grandmother, and EMS, intravenous (IV) access was obtained, and weight-based dosing of diphenhydramine was administered.

During the observational period in the ED the patient remained inconsolable, most likely secondary to pain. He continued to keep his mouth in an open position with occasional episodes of gagging and drooling. The patient was witnessed vomiting colorless emesis, with occasional blood-streaked emesis. He subsequently had a significant increase in the amount of drooling. Upon a more thorough inspection of the oropharyngeal cavity, the patient was noted to have erythema and ecchymosis on the inside of the right upper and lower lip regions.

Due to suspicions about the provisional diagnosis, the attending physician decided to perform a pH test of the emesis, using a pH strip ordinarily used for ophthalmological pH testing. The pH strip indicated a pH of 9. The focus of the patient’s care plan immediately shifted to the evaluation of a patient with a caustic ingestion. After consultation with the pediatric intensive care unit (PICU), a presumptive plan for nil per os (NPO) and endoscopy were discussed. The patient was transferred to an affiliated tertiary care facility.

The admitting PICU team considered the initial diagnosis of allergic reaction more likely and therefore proceeded with treatment for an allergic reaction, which did provide some improvement in the patient’s oral labial edema. Approximately two days after the patient’s initial presentation to the ED, he underwent an upper endoscopy because he continued to refuse to eat or drink. Endoscopy revealed grade 2b findings (see Table) in the esophagus and gastric cardia. Based on the endoscopic findings the diagnosis was changed to a caustic ingestion. The patient remained hospitalized, was placed on NPO and received a peripherally inserted central catheter for total parenteral nutrition; he was given IV fluids, placed on a proton pump inhibitor, sucralfate, and ranitidine. Antibiotic prophylaxis was given with ampicillin/sulbactam and steroids were started. Over his hospital course, his diet was slowly advanced. He was eventually discharged home after one week when tolerating a soft diet. Although the family initially denied potential ingestion, it was later discovered that the patient was a victim of non-accidental trauma, where one of the family members forced him to ingest an industrial bleach compound in an attempt to stop the child’s crying.

## DISCUSSION

In the Western world, alkaline material accounts for most caustic ingestions injuries.^1^ Esophageal injury can begin within minutes and may continue for hours. These burns can lead to chronic complications such as esophageal strictures, reported between 2%–63% of cases, and increased incidence of esophageal cancer, reported in 18%–46% of cases.^3^–^7^

Acids and alkalis create tissue damage differently. Acids usually cause coagulation necrosis leading to eschar formation and limited deep tissue penetration. Alkalis tend to cause liquefactive necrosis and thrombosis in blood vessels leading to the destruction of deeper tissues. Both acids and alkalis can penetrate the esophageal wall rapidly and can cause full-thickness damage. The degree of severity of gastrointestinal injury due to caustic ingestion is associated with several factors including the pH of the agent, physical state (solid, liquid, or powder), tissue exposure time, and quantity or concentration of the substance. The pH of standard liquid industrial bleaches, detergents, and phosphates ranges from 9 to 11. Gastrointestinal injury of caustic origin is linked to high rates of morbidity and mortality.^8^

CPC-EM Capsule

What do we already know about this clinical entity?

The use of pH paper has been validated measuring intragastric pH, but little is known about its usefulness in measuring the pH of emesis, especially in suspected ingestion.

What makes this presentation of disease reportable?

This is the first report of ophthalmic pH paper being used to diagnose an occult caustic ingestion.

What is the major learning point?

Early diagnosis of caustic ingestion and esophageal injury helps to initiate appropriate therapy, anticipate airway intervention, and decrease overall morbidity.

How might this improve emergency medicine practice?

pH paper should be stocked in every ED and is a quick, simple, and inexpensive test that can lead to a rapid diagnosis and faster implementation of treatment.

The pH of gastric acid in the stomach of children is typically less than 4.0.^9^ One study measured regional postprandial differences in pH within the stomach and gastroesophageal junction on various types of foods. After 27 hours of monitoring the highest pH at any region was 5.4.^10^ Emesis should yield a moderately acidic result. If this emesis is tested with a pH paper, it should indicate an acidic state (pH <7). However, when high quantities or concentrations of basic substances are exposed to gastric acid, it is proposed that the pH should be buffered (pH ~7) or basic (greater than 7) (Image). pH-tested emesis with a neutral or basic pH should raise concern for possible alkaline ingestion; however, an acidic pH does not rule out an alkaline ingestion. Litmus paper has been shown to be sensitive and specific for measuring intragastric pH when compared to gastric samples taken from a nasogastric pH probe.^11^ Although easy to use, clinicians should be aware that pH measurement errors can occur from excessive wetting of the paper, small sample sizes, allowing drying of the paper, and using expired litmus paper. Some reports advocate the use of a control when doing any litmus test; however, this would be difficult to do with emesis.^12^

Pediatric patients who are unable to give a reliable history and present with emesis and oral burns from an unknown ingestion or from a substance known to cause esophageal or gastric injury, should have an esophagogastroduodenoscopy (EGD) performed early. In this case, as well as other caustic ingestions, early EGD is considered crucial and recommended within the first 12–48 hours.^13^ Endoscopic classification is important in determining prognosis and management. A grading system has been adopted based on visualization during endoscopy and helps to predict clinical outcomes (*Table*).^14^ Huang et al. reported that all patients with grade two and three injuries developed esophageal strictures.^15^ Early esophageal dilatation is recommended to improve the outcome of esophageal injury and reduce the number of patients developing esophageal stricture.^16^

Early ED management is important not only for acute intervention but also for helping establish a treatment course that can ultimately lead to fewer long-term complications. Early management includes airway assessment and hemodynamic stabilization. For patients with persistent vomiting and severe oropharyngeal injury, airway protection is vital, and early intubation should be considered. Fiberoptic laryngoscopy can allow direct visualization versus a “blind” intubation, which increases the risk of additional injuries. Blind placement of nasogastric tubes is not recommended due to the risk of perforation or additional injury.

Late ED management is primarily conservative. Gastric lavage, inducing emesis and pH neutralization are contraindicated. Use of corticosteroids and antibiotics remains controversial.^17^ Literature analysis dating back to 1956 has not found any benefit for steroids preventing stricture formation.^18^ One small study in 2014 showed a large difference in stricture development in patients with grade 2b burns, between a control group receiving ceftriaxone and ranitidine and treatment group receiving methylprednisolone, ceftriaxone and ranitidine, with stricture development in 30% vs. 10.8% of patients, respectively^19^. A small prospective study showed IV omeprazole might effectively be used in the acute phase treatment of caustic esophageal injuries.^20^

The most common complication after caustic ingestions includes gastric outlet obstruction, esophageal stricture formation and an increased risk of esophageal carcinoma. It is estimated that strictures will form in 3–57% of patients who ingest caustic substances and in nearly 100% who develop circumferential burns.^3^,^7^,^21^ Esophageal neoplasms (squamous cell carcinoma and adenocarcinoma) may develop later in life at a rate 1,000–3,000 times higher than the general population.^22^ These life-changing comorbidities make early diagnosis and intervention critical to the improved quality of life post-ingestion for these patients.

## CONCLUSION

Caustic ingestion is a race against time. Early diagnosis of caustic ingestion and esophageal injury is imperative in initiating appropriate therapy and decreasing overall morbidity. There is limited literature regarding pH testing of unknown ingested substances and its diagnostic value. There has been even less discussion on testing the pH of emesis in the setting of suspected ingestion. The use of pH paper on emesis is a practical way to help the confirm suspicion of caustic ingestion. It is not practical in cases of suspected acidic ingestion but can be beneficial if concerned about alkaline agents. These pH paper strips should be stocked in every ED. They offer a quick, simple, and inexpensive test that should be added to our armamentarium as emergency physicians.

## Figures and Tables

**Image:**
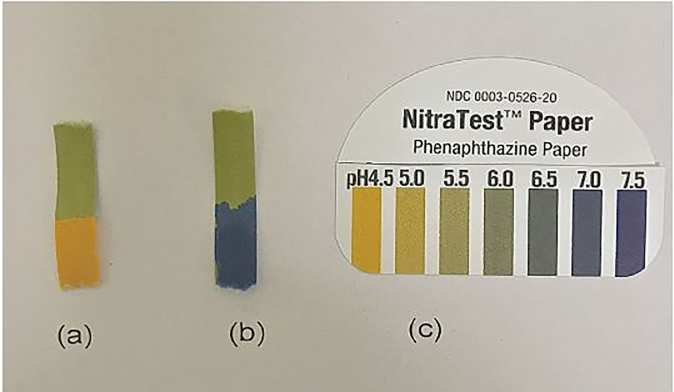
NitraTestTM Paper tested on emesis of a pediatric patient (a), emesis of the same pediatric patient mixed with industrial drain cleaner comprised of 90% emesis and 10% drain cleaner (b), and control strip (c).

**Table:** Endoscopic grading of esophageal injuries (reproduced from Zargar et al,^2^)

Grade	Features
Grade 0	Normal mucosa
Grade 1	Superficial mucosal edema and hyperemia
Grade 2A	Superficial erosions, exudates and ulcerations
Grade 2B	Deep discrete or circumferential ulcerations
Grade 3A	Small scattered areas of focal necrosis
Grade 3B	Extensive necrosis
